# Student challenges and successes in integration of planetary health in medical education: a mixed methods analysis

**DOI:** 10.3389/fpubh.2025.1593332

**Published:** 2025-07-24

**Authors:** Kanika Malani, Emily Yamron, Kate Tokareva, Taylor Brewer, Alexander Northrop, Lauren Franklin, Kyle Denison Martin

**Affiliations:** ^1^Yale New Haven Hospital, New Haven, CT, United States; ^2^Albert Einstein College of Medicine, Bronx, NY, United States; ^3^School of Medicine, University of Washington, Seattle, WA, United States; ^4^The George Washington School of Medicine and Health Sciences, Washington, DC, United States; ^5^Columbia University Vagelos College of Physicians and Surgeons, New York, NY, United States; ^6^School of Medicine, Keele University, Staffordshire, United Kingdom; ^7^Warren Alpert Medical School of Brown University, Providence, RI, United States

**Keywords:** planetary health, climate health, medical education, medical curriculum, climate change

## Abstract

**Introduction:**

This study evaluates challenges and successes faced by medical students leading planetary health (PH) curriculum reform and tracks changes over time.

**Methods:**

The Planetary Health Report Card (PHRC) is a standardized, metric-based tool for assessing PH education in medical schools. In October 2023, a survey was emailed to 132 medical students across 52 US institutions who had served as primary contacts for their school’s most recent PHRC between 2020 and 2023. The survey explored challenges and successes in integrating PH into the curriculum. A total of 31 students from 17 US states responded. All respondents were invited to follow-up Zoom interviews, with 11 completing them. Survey and interview data were analyzed using descriptive statistics and directed content analysis, while longitudinal PHRC data were assessed using Wilcoxon signed-rank tests.

**Results:**

Students are primary stakeholders in PH integration (*N* = 28, 90%). While other stakeholders were perceived as generally supportive, surveyed students felt these groups were less likely to lead PH curricular reform and may lack confidence in delivering PH content (*N* = 7, 70.0%). Interviewees (*N* = 11) noted various obstacles and suggested potential solutions to improve PH integration into medical curricula.

**Discussion:**

Medical schools should prioritize developing longitudinal, course-specific PH education, supported by both individual institutions and national frameworks and licensing organizations.

## Highlights

- Identify challenges and successes medical students face in integrating planetary health into curricula.
- Evaluate changes in planetary health integration in United States medical curricula from 2020 to 2023.
- Identify strategies to increase planetary health integration in medical curricula at various levels.

## Introduction

Planetary Health (PH) is defined as the reciprocal relationship between the health of human civilization and the state of the natural systems upon which it depends (1). It is also a “solutions-oriented, transdisciplinary field and social movement focused on analyzing and addressing the impacts of human disruptions to Earth’s natural systems on human health and all life on Earth (2)”. The Canmore Declaration, a statement of principles for planetary health, further underscores the interconnectedness of people, places, and the planet, advocating for a holistic approach to health and sustainability (3). Related frameworks, including One Health and EcoHealth, share this systems-oriented perspective. One Health takes an integrated approach to sustainably balance and optimize the health of people, animals, and ecosystems, recognizing their interdependence (4). EcoHealth similarly unites natural and social sciences to manage and understand ecosystems and the links between human, animal, and environmental health (4). While each of these frameworks varies in scope and emphasis, they collectively highlight the need for transdisciplinary collaboration to address growing environmental and health challenges. The Planetary Boundaries framework—a key precursor to PH—further grounds this field by defining nine critical Earth system processes (climate change, biosphere integrity, land system change, freshwater use, biogeochemical flows, ocean acidification, atmospheric aerosol loading, stratospheric ozone depletion, and introduction of novel entities) that regulate the stability and resilience of the planet (5). These boundaries establish safe environmental thresholds within which humanity can thrive, but many are already transgressed or at high risk due to escalating human pressures (5).

As these boundaries are crossed and environmental threats such as climate change and biodiversity loss intensify, it becomes increasingly urgent to incorporate PH into medical education (6–8). This integration equips future medical professionals not only to recognize and manage the health impacts of environmental change, but also to engage in advocacy, lead systemic interventions, and collaborate across disciplines to promote PH. Given that the healthcare sector contributes 4.4% of global net greenhouse gas emissions, primarily through its supply chain, physicians are uniquely positioned to lead efforts in reducing healthcare’s carbon footprint (9). Their trusted role in society further strengthens their ability to influence sustainable healthcare practices and advocate for systemic change. However, despite the medical field’s potential to drive these efforts, medical curricula lacks the integration of PH.

Globally, the integration of PH into medical education varies, but it broadly aims to cultivate values, behaviors, knowledge, and skills for understanding, managing, and mitigating the health consequences of climate change. In 2020, the General Medical Council, which independently regulates doctors in the United Kingdom, published learning outcomes for sustainable healthcare, mandating that medical schools align their teaching with these standards (10). Similarly, in April 2023, the Association of Faculties of Medicine of Canada issued a Declaration on PH, committing signatories—including 15 of Canada’s 17 medical schools along with medical schools and health organizations from around the world—to “immediately work to align our [their] healthcare schools with the Planetary Health Education Framework, providing common foundational principles, competencies, and language to prepare future healthcare professionals to both mitigate further environmental degradation and to lead and contribute to adaptation and resilience strategies” (11). In the United States (US), while the American Medical Association (AMA) has called for the integration of “the health implications of climate change into the spectrum of medical education,” bodies governing medical education have yet to establish clear requirements or standards for PH content (12). Nevertheless, global efforts to implement PH education are increasing, largely driven by student advocacy; medical students have played key roles in promoting curriculum reform, designing coursework, and leading co-curricular initiatives (13–18).

Two prominent national and international organizations advancing student-led PH efforts are the Planetary Health Report Card (PHRC) and Medical Students for a Sustainable Future (MS4SF) (16, 19). The PHRC, created in 2019 by medical students at the University of California, San Francisco, is a standardized, metric-based tool for evaluating PH offerings in medical schools. It assesses gaps in curricula, identifies areas for improvement, and promotes collaboration in setting educational goals (16). Beyond curriculum evaluation, the PHRC examines co-curricular opportunities and campus sustainability (13). To our knowledge, it is the only standardized tool in the US used to evaluate PH curricula. MS4SF, also founded in 2019, is an organization dedicated to uniting medical students invested in planetary health and equipping them with resources to advocate for change (19). Its initiatives span advocacy, curriculum reform, research, and climate-smart healthcare (19). Given the structure of undergraduate medical education in the US—divided into pre-clerkship and clerkship phases (20–22)—there is significant opportunity for integrating PH concepts across both these stages of medical training (23).

Despite the progress made by these PH organizations in supporting curriculum changes across numerous health professional schools (14, 24, 25), a comprehensive tool to evaluate the PH curricular development process is lacking. To address this gap and inform future curricular improvements, the specific aims of this study were to: (1) develop and implement a novel, standardized survey tool to assess stages of medical curricular development where student advocates encounter challenges in integrating PH content; (2) conduct in-depth interviews to further explore these challenges; and (3) evaluate whether these student-reported challenges align with trends in PHRC scores.

## Materials and methods

### Overall study design

A mixed-methods approach was used, incorporating surveys, qualitative interviews, and analysis of publicly available PHRC data. The Brown University survey research center oversaw the development of the survey and interview questions, ensuring the language used was clear and inclusive for all participants. Questions were designed by the study authors, who have experience implementing PH curricula at six institutions, to ensure content validity. The study adhered to the Strengthening the Reporting of Observational Studies in Epidemiology (STROBE) cross-sectional checklist (see Supplementary file) (26). Informed written consent was obtained from survey participants. Informed written and verbal consent was obtained from interviewees.

### Ethics statement

This study was deemed to be a quality improvement study and not human subjects research by a local research application form through Brown University and the National Institute of Health Human Subjects Research Decision Tool (27, 28). As a quality improvement project exempt from IRB approval and anonymous in nature, formal verbal or written consent was not required.

### Participant recruitment

Each year, medical student groups completing the PHRC publicly identify a primary contact individual and their email address. Students who served as primary contacts for 52 US-based medical schools’ PHRCs between 2020 and 2023 were invited to participate in the study through an anonymous survey link that was sent out to the eligible participants (*n* = 132) twice. PHRC participants outside the US were excluded because international medical programs differ from US programs in terms of structure, program length, and governance. Additionally, the majority of PHRC data is derived from US institutions. By focusing on US schools, we were able to ensure a more consistent framework for examining PH integration. Survey data from 31 primary PHRC contacts were collected from October 11, 2023, to October 31, 2023. Survey respondents who wished to participate in a subsequent Zoom interview (*n* = 24) were given the option to anonymously provide their contact details through a separate, unlinked form, ensuring their survey responses remained confidential. Of those, 11 ultimately completed the interview.

### Survey design

Survey questions were designed to understand the challenges and successes students experienced when integrating PH into their medical school curricula. As such, survey questions addressed various aspects of curricular development including stakeholder involvement, stages of development and integration, resources used, and institutional demographics. A Likert scale was utilized for questions assessing common obstacles participants faced in integrating PH into curricula. The full survey was created on Qualtrics (28) and is available as a Supplementary file. Due to the already limited sample size of primary PHRC contacts, the survey was not piloted prior to distribution.

### Interview design

One-on-one Zoom interviews were conducted with interested participants from November 16, 2023 to December 1, 2023. Interview questions, developed through iterative discussions with 4 study members (KM, EY, KT, LF), explored the current state of PH in curricula, the process and stakeholders involved in its development, challenges encountered during this development, the uniqueness of these challenges, resources needed for PH integration into curricula, opinions on the adequacy of current PH inclusion in curricula, and views on the ideal level of PH in the curricula. The interviews were semi-structured and discussion-based. They were conducted by one study member (KM or KT). No compensation was offered. All interviews were recorded and transcribed verbatim using Transkriptor (29).

### Survey data analysis

Survey data on PH curricular integration and challenging and successful aspects of this curricular development were summarized using descriptive statistics. Additional survey data were analyzed in R for Statistical Computing (R version 4.2.2) (30).

### Interview data analysis

Interview responses were coded using directed content analysis by two study members (KM & KT). Through independently reviewing the audio recording and transcription of one interview, both members generated a list of initial themes. They then discussed the themes for this interview to collaboratively refine the coding system until consensus was reached. The refined coding system was used to code the remaining 10 interviews with discrepancies resolved through further discussion and revision of coded themes. A profile of the interviews’ thematic categories and their relative frequencies was created. The study team debriefed after completing all interview coding to clarify and verify overarching themes. Quotations from participants were used to illustrate each theme, with brackets added to provide relevant context and understanding. The use of brackets was reviewed to ensure the final quotations preserved the integrity and meaning of participants’ original responses.

### PHRC data analysis

A database of publicly available PHRC data from 2020 to 2023 was created to analyze PHRC curricular scores. The 2023 version of the PHRC included 22 questions with a cumulative curricular score out of 69 possible points (31). All US medical schools with more than one PHRC report card were included in the analyses in order to assess changes in curricular scores over time. To account for variations in PHRC report card structure from 2020 to 2023, the percentage of curricular points a school received from the total available curricular points was used. Report years were standardized with the first report produced designated as Report Year 1 (RY1), followed by RY2, RY3, and RY4 since schools began participating in the PHRC in different years. Wilcoxon signed-rank tests were used to compare each school’s median curricular scores between their first and last PHRC, as well as yearly differences (e.g., RY2 vs. RY3). Of note, there were insufficient schools in RY4 to evaluate the data with a Wilcoxon signed-rank test.

## Results

### Survey

Of the 132 students contacted, 24% (*n* = 31) completed the survey. Respondents represented medical schools across all US census regions. Among the 31 respondents who provided demographic information, 77% (*n* = 24) attended schools with a class size of 100–200 students, and 52% (*n* = 16) attended public institutions (Supplementary Table S1).

90.3% (*n* = 28) of respondents reported that students were the primary stakeholders leading curricular reform. 90.3% (*n* = 28) indicated that at least some level of PH had been integrated into their institution’s curricula. Additionally, 22.6% (*n* = 7) noted their school had integrated PH content into curricula but lacked plans to add more. In contrast, 54.8% (*n* = 17) reported their school was developing plans to update, review, and/or add PH content into their curricula (Table 1).

**Table 1:** Stakeholders contributing to planetary health curricular reform and levels of planetary health (PH) integration attained by medical schools according to survey respondents (*n* = 31).

Stakeholder^a^ & Stage of PH Integration	n (%)
Most Active Stakeholders in PH Curricular Reform^b^	
Students	28 (90.3)
Lecturers/Instructors	1 (3.2)
Deans	1 (3.2)
Course Directors	1 (3.2)
Stage of PH Integration into an Institution’s Medical Curricula	
Not started	0 (0)
Discussed, not Implemented	3 (9.6)
Have integrated without plans to update/review/add	7 (22.6)
Have integrated with developing plans to update/review/add	17 (54.8)
Have integrated with existing plans to update/review/add	4 (12.9)

a Stakeholder refers to individuals or groups with a vested interest in PH curricular reform.

b “Curriculum committee,” “community members,” “other,” and “currently no one leading curricular reform” were also included as options, but were not selected by any survey respondents.

**Figure 1:**
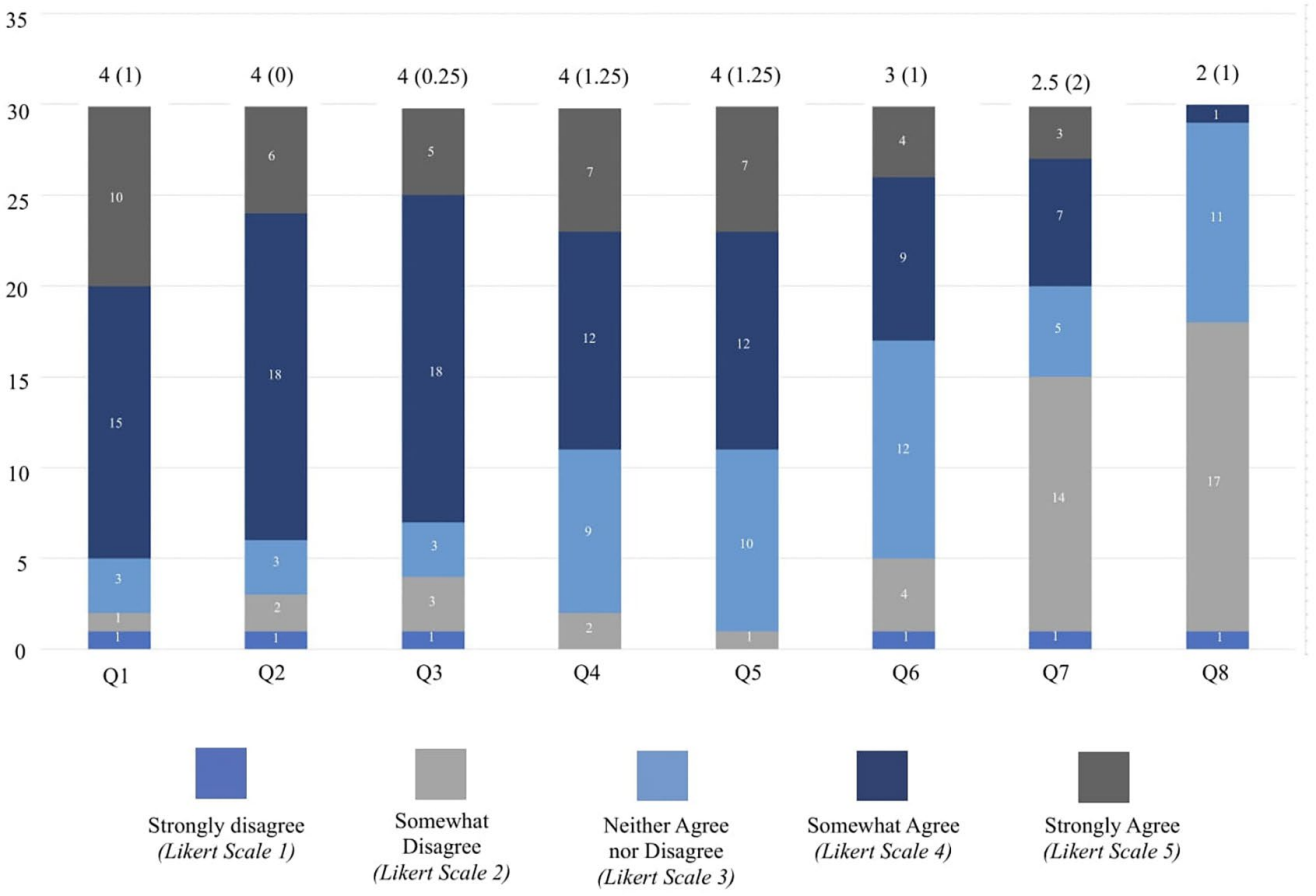
This figure shows the distribution of responses to statements about planetary health (PH) integration in medical school curricula according to survey respondents (*n* = 31). A 5-point Likert scale ranging from strongly disagree (1) to strongly agree (5) was used to show the distribution of responses to statements regarding PH integration in medical curricula. For each item, median (IQR) are reported above the corresponding bar. Number of respondents choosing each option are denoted along the y-axis. The statements include: (1) The people leading curricular development are able to identify places to add PH content into your existing curriculum OR ways to modify your existing curriculum to integrate PH; (2) Administrators express support for integrating PH content; (3) Course directors express support for integrating PH content; (4) Lecturers/instructors express support for integrating PH content; (5) The people leading curricular development are able to find resources to integrate PH content. (e.g., slides, case conferences, learning objectives, curricular outlines/syllabi, elective coursework syllabi); (6) The existing resources available (e.g., MS4SF curriculum guide and/or the CRHE resources) can be adapted into your institution’s curriculum without much effort; (7) There is enough time in the curriculum to teach PH content; (8) Instructors feel confident teaching PH content.

The percentage of respondents who agreed either somewhat or strongly with the subsequent statements are as follows: “people leading curricular development can identify places to add or modify PH content into the existing curriculum” (*n* = 25, 80.6%), “people leading curricular development can find resources to integrate PH content into curriculum” (*n* = 19, 61.3%), “existing resources can be adapted into the institution’s curriculum without much effort” (*n* = 13, 41.9%), “instructors feel confident teaching PH content” (*n* = 3, 9.7%). The majority agreed that the following stakeholders express support for integrating PH into curricula: administrators (*n* = 24, 77.4%), course directors (*n* = 23, 74.2%), lecturers/instructors (*n* = 19, 61.3%; Figure 1).

Students reported using a variety of sources to acquire PH content to be integrated within the curricula. Resources created within individual institutions were most utilized (*n* = 20, 64.5%), followed by resources created at other institutions (*n* = 14, 45.2%), resources from climate organization repositories (*n* = 12, 38.7%), and other resources (*n* = 6, 19.4%).

### Interviews

Of the 22 participants who expressed interest in a follow-up interview in their initial survey response, 11 scheduled interviews to further discuss their experiences with implementing PH in their respective institution’s curriculum. Participants were primarily from medical schools located in the western (*N* = 4, 36%) and northeastern (*N* = 5, 45%) US census regions (groupings of states based on geographic divisions according to the US Census Bureau). Most participants had been involved in PH work for 2 to 3 years (*N* = 9, 82%) and were in their third or fourth year of medical school (*N* = 8, 73%). Interview themes and their frequencies, along with quotations from participants, are presented in Table 2.

**Table 2:** Qualitative interview themes with reported frequencies and quotes from interviewees regarding planetary health (PH) in medical curricula (*n* = 11).

Current PH framework within medical curricula	Quotations
Primarily within pre-clerkship/pre-clinical medical curricula (*n* = 10, 91%)	“Currently, our planetary health content is essentially limited to the first two years of school. After that we have a waste training prior to starting clinicals [clinical rotations], but nothing else embedded in our curriculum.”
Standalone (*n* = 8, 73%)	“Planetary health affects so many different areas of medicine and having a standalone lecture or two [like we do] is not sufficient.”
Main PH curricular integration advocacy efforts	
Student-led (*n* = 11, 100%) through content development (*n* = 11, 100%) and institutional advocacy (*n* = 6, 55%)	“We got student signatures to show that this was really important to us so the admin could see and the [planetary health] class was added.”
Obstacles encountered in PH integration into medical curricula	
Lack of space in medical curricula (*n* = 10, 91%)	“A lot of course directors are interested in theory, but practically, feel like there is not a lot of time to incorporate new material because there is so much to teach.” “As more schools cut down on pre-clinical time, I would not be surprised if other schools face this [obstacle] as well.”
Lack of national board accountability (*n* = 8, 73%)	“Students and schools are not incentivized to learn and incorporate planetary health into curriculum because it is not included in national exams. If there was more national accountability, planetary health would become more of a priority.” “People are already disgruntled with the discordance between what’s on STEP [standardized exams] and what’s in our own materials.”
Lack of faculty bandwidth (*n* = 7, 64%)	“Administration and faculty are supportive of change, but do not seem to be able to actively lead it, resulting in students bearing the burden of solving this issue.” “We do not know how to write a curriculum for them—from the student side, inexperience with developing the curriculum since we are students. And faculty bandwidth—they are already spending lots of energy doing the basics, they do not know if they can fit it [planetary health] in.”
Desired PH components in medical curricula	
Longitudinal (*n* = 10, 91%)	“[We want to work on] making planetary health a curricular theme and weaving planetary health into existing material rather than creating a whole new session.” “Ideally there is [planetary health] content that builds out throughout the clinical and pre-clinical curriculum.”
Incorporation into clerkship/clinical curricula (*n* = 8, 73%)	“We have a session on waste in the operating room in surgery [rotations] and a session on eco-anxiety in psychiatry [rotations].”
Required for all students (*n* = 8, 73%)	“Planetary health is becoming an increasingly important part of medicine. We need to train students who are well-equipped to treat the many patients who are impacted by planetary health changes.”
Strategies to enhance PH integration into medical curricula	
Additional resources (*n* = 9, 82%)	“I know there are some resources out there to help include planetary health into curriculum, but it would be helpful if we had more resources to draw from so we can pick which ones best fit our school’s curriculum-whether it’s a lecture or case-based scenario or discussion.”
Examples and experiences of successful integration of PH within medical curricula (*n* = 6, 55%)	“[We should] get examples and inspiration for how we [students as a whole] are doing this; it’s always great to use other people’s examples and bounce off of that.”
Administrative support (*n* = 6, 55%)	“[Ideally, we want a] faculty/administrative member whose sole role is to integrate planetary health with curriculum and interact with students that are interested in this.”

a. Medical education in the United States is divided into two main phases: pre-clerkship/pre-clinical curriculum and clerkship/clinical curriculum. The pre-clerkship/pre-clinical curriculum phase spans approximately the first 2 years of medical school and focuses on foundational knowledge and early clinical skills. The clerkship/clinical curriculum phase covers approximately the last 2 years of medical school and involves direct patient care under supervision.

Discussions on the current framework of PH in interviewees’ schools revealed that the structure tends to be standalone (*n* = 8, 73%) rather than longitudinal, with primary integration in pre-clinical years (*n* = 10, 91%). Interviewees also highlighted that students are the primary advocates for PH curricular development (*n* = 11, 100%), mainly through content development (*n* = 11, 100%) and internal advocacy (*n* = 6, 55%). Common obstacles to PH integration include lack of space in medical curricula (*n* = 10, 91%), lack of accountability from medical boards (*n* = 8, 73%), and lack of faculty bandwidth (*n* = 7, 64%). All interviewees expressed that their institution’s curriculum does not include adequate PH content (*n* = 11, 100%). Interviewees envisioned ideal PH inclusion in medical curricula as involving a longitudinal structure (*n* = 10, 91%) integrated into both pre-clinical and clinical years (*n* = 8, 73%), with mandatory participation for all students (*n* = 8, 73%). Interviewees also identified the following as helpful in furthering PH curricular development: additional PH curriculum resources (*n* = 9, 82%), examples of successful PH integration experiences (*n* = 6, 55%), and administrative support (*n* = 6, 55%).

### Planetary health report card

**Figure 2:**
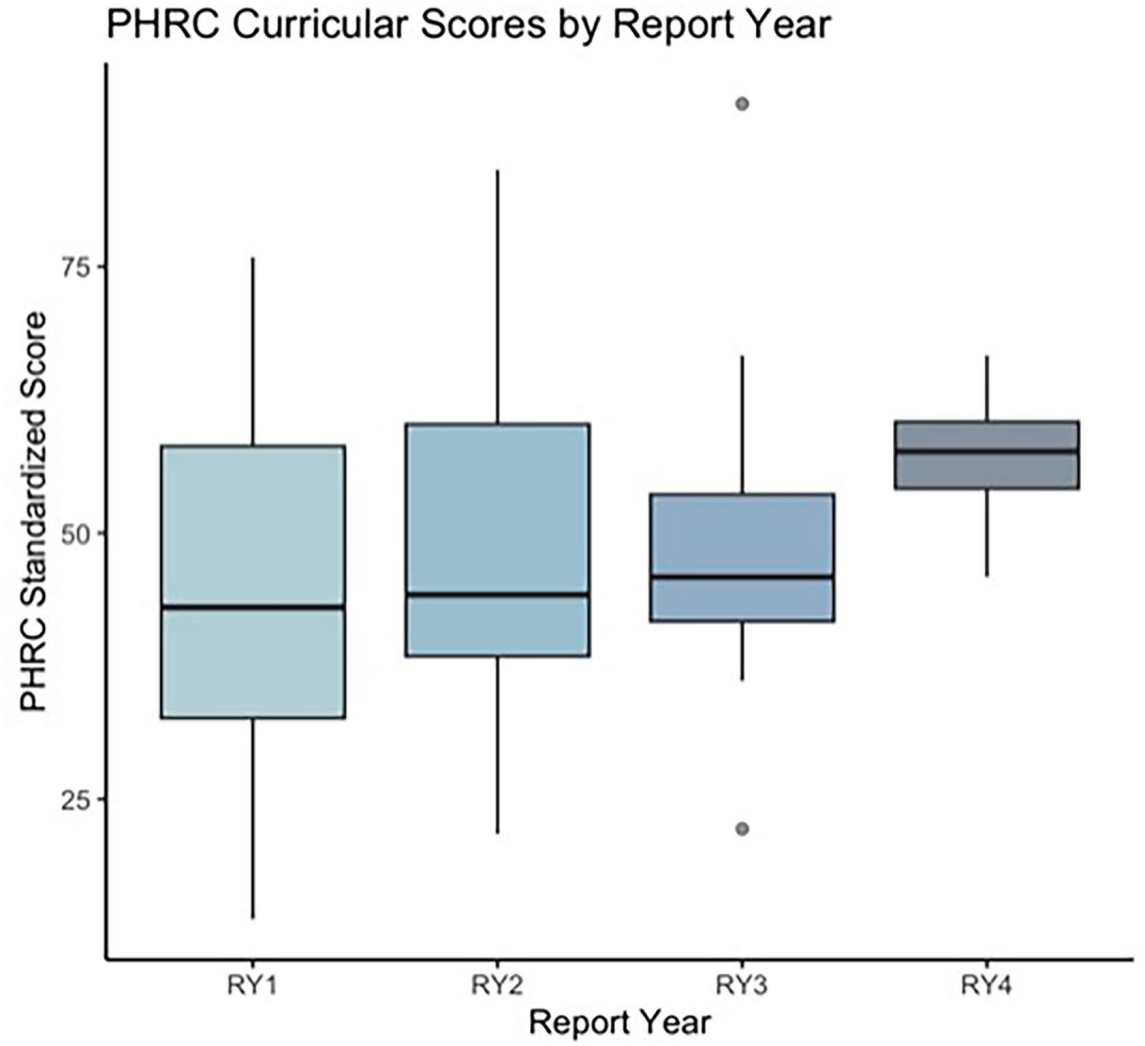
This figure shows aggregated Planetary Health Report Card (PHRC) scores across United States medical schools by report year (RY; *N* = 32). Box and whisker plots were used to show aggregated and standardized PHRC scores for medical schools across the United States by RY.

32 US medical schools met the criteria for analysis inclusion. The curricular scores during the first report year (RY) were lower (Median: 43.0; IQR: 32.6–58.1) than the last RY (Median: 57.6; IQR: 54.2–60.0) at the aggregate level (z = −2.78; *p*-value = 0.005). When comparing individual schools’ first and last RY, the median difference between the two RYs was +5.7. Moreover, multiple comparisons showed an increase in PHRC median scores from RY1 to RY2 (z = −2.08; *p*-value = 0.04) and from RY2 to RY3 (z = −2.22; *p*-value = 0.03). There were not a sufficient number of schools (*N* = 4, 12%) on RY4 to evaluate the data with a Wilcoxon signed-rank test. Grouped descriptive data illustrated similar improvements in curricular scores over time across all report years (Figure 2).

## Discussion

In this mixed-methods national study assessing the challenges and successes medical students face in integrating PH into curricula, we find that students consider themselves most actively involved in spearheading this curricular reform among all stakeholders. While other stakeholders—including instructors and school deans—are viewed as generally supportive, students report that these groups are less likely to be leading the change and may lack confidence in delivering PH content. Additionally, while places to add PH content to curricula are identifiable, respondents report lacking easily accessible and modifiable resources to facilitate this process. Survey and interview data both demonstrate the widespread attitude among participants that the current level of PH integration in medical curricula is insufficient. This sentiment is reflected in the setting of a majority of respondents reporting that their schools have standalone PH content and lack existing plans to advance PH integration through methods like restructuring into a mandatory, longitudinal format, which respondents prefer. While respondents feel broad curricular changes are needed, PHRC data suggest that successful reform in PH integration into medical curricula is underway.

The challenges to integrating PH into curricula identified in this study mirror those seen in previous research (24, 25, 32). These challenges can be categorized into three interconnected levels: course-specific, institutional, and national. While there are published peer-reviewed PH learning objectives (33) and PH content databases, like Climate Resources for Health Education (CRHE) (34), which offers resources like lecture objectives and sample materials, these still require customization to fit the unique needs of each institution’s courses. Additionally, one of the greatest obstacles identified in our interviews and previous studies (35, 36) was the lack of space and time within medical curricula for additional content, especially given that many schools are now shortening pre-clinical curricula. To address this, interviewees proposed integrating smaller portions of PH content across multiple courses in a longitudinal manner. To concurrently address these course-specific difficulties, we propose collaborating with organizations like CRHE to streamline their existing resources for easier integration into time-limited curricula. Moreover, establishing a team of education specialists within such organizations, akin to existing groups like the American Association of Medical Colleges (AAMC) Curriculum Committee (37) and the AMA Council on Medical Education (38), could support the customization of these resources for individual institutions. However, it is essential to note that this challenge of PH integration extends beyond individual courses and reflects a more comprehensive institutional curricular issue as well.

At the institutional level, respondents felt that many faculty and administrators voice support for PH integration, but have limits, either in terms of knowledge or time, that hinder their capacity to offer substantial assistance. This aligns with previous studies, including one on sustainable healthcare education in UK medical schools, where a lack of educator confidence was cited as a barrier to curriculum integration (39). Similarly, prior research emphasized the knowledge gap among educators as a barrier to teaching PH effectively (35, 36). To address this institutional challenge, we recommend establishing dedicated medical faculty positions in PH, similar to those at Harvard’s Center for Climate, Health, and the Global Environment (40) and Stanford’s Human and Planetary Health faculty roles (41). We also advocate for closer collaboration between medical and public health schools, many of which offer specialized planetary health tracks (42). Additionally, interviewees suggested that sharing “success stories” from other institutions could inspire and guide future efforts. This could be facilitated through dissemination of these successful endeavors through publications, curriculum guides, and educational conferences (23, 43–45). Finally, discord between school curricula and standardized board exam content was identified as a significant barrier to PH integration, which represents an institutional and national challenge.

Currently, national United States Medical Licensing Examinations (USMLE), including STEP 1, 2, and 3, currently do not adequately emphasize PH despite robust evidence of climate’s impact on health (46–48) and the alignment of PH content with established STEP objectives in population health, legal/ethical issues, and patient safety (49, 50). Interviewees noted that the disconnect between curriculum content and board exam relevance may contribute to a lack of institutional impetus for further PH integration. One potential approach to addressing this gap could involve incorporating PH experts on USMLE committees (49, 50) and standardizing learning goals nationwide—a strategy that might draw on international frameworks, such as the Association for Medical Education in Europe’s objectives for environmentally accountable medical curricula (51). Standardizing these goals could also help reduce variability across institutions and ease the burden on those currently developing PH materials independently. Although these considerations extend beyond the immediate scope of this study, they offer a promising direction for future research and policy development.

Despite challenges in enhancing PH in medical curricula, the PHRC data analysis reveals an increase in PHRC curricular scores over time, both by individual school and in aggregate. This suggests improved PH integration in medical curricula since 2020, a trend also supported by data from the AAMC, which indicates a rise in climate change-related topics in undergraduate medical education in recent years (52). These improvements are beginning to address a significant gap that medical students perceive in their education (53). With further changes, as outlined above, this gap can be further narrowed, better equipping doctors to confront the growing health threats posed by climate change.

A key strength of this research is its provision of insights directly from US medical students involved in planetary health (PH) curricular reform, an underrepresented perspective in current literature. Given that the healthcare sector is the fifth-largest global carbon emitter and the US has the highest per capita carbon emissions (9), this US-based study is particularly significant in evaluating PH integration. Additionally, while previous studies on barriers to PH integration have primarily focused on international medical educators’ perspectives (39, 54), this research offers a unique contribution from a US standpoint.

However, this study has several limitations that may impact the generalizability of our findings. First, the survey tool used lacks external validation, though its structure and questions were reviewed by qualitative data personnel to ensure content relevance, clarity, and alignment with the study’s objectives. Secondly, a potential bias in this study stems from the survey being exclusively delivered to students, which may influence their perception of themselves as the primary stakeholders in PH curriculum integration. This viewpoint is largely shaped by students’ direct experiences with the medical curriculum, providing them with firsthand insight into gaps in PH education and the urgent need for reform. We intentionally focused on students because they are often the front-line advocates for curricular change, a notion supported by previous research emphasizing their critical role in driving reform. While including faculty, administrators, and other stakeholders within PH education could have enriched the data by offering additional perspectives on institutional challenges or barriers to integration, our aim was to capture the voices of those most immediately affected by the curriculum. As such, broadening the survey population was beyond the scope of this study. Additionally, the sample may not represent all US medical schools or international institutions, as it excludes students from schools outside the US and focuses specifically on participants in the PHRC. Since the PHRC is an opt-in initiative, it captures only those students interested in PH integration, which may not reflect the experiences of students at institutions not involved in the PHRC. Nevertheless, those engaged in the PHRC are likely to be at the forefront of PH curricular reform, making our sample representative of students actively leading such efforts in the US, despite its relatively small size. Despite being US-based, this study is valuable in filling the gap in research on PH curricular integration broadly.

### Future directions

Surveying additional stakeholders, such as faculty and deans, was beyond the scope of this work. However, exploring their attitudes is crucial for understanding other barriers to successful PH integration. Future efforts should also prioritize the development of interprofessional and transdisciplinary PH curricula that reflect the complex, systems-based nature of the PH crisis. These curricula must include expertise from outside traditional medicine, such as veterinary medicine, climate science, urban planning, and environmental policy, to ensure students understand both the causes and relevance of the PH crisis. Including faculty experts from these diverse fields not only enriches the curriculum but also helps address capacity limitations faced by traditional medical faculty in delivering comprehensive PH content. Collaborative learning opportunities, such as joint coursework, case-based learning, and simulation exercises co-developed and co-taught across disciplines, should be paired with interdisciplinary capstone projects and community-engaged initiatives. Involving students in learning alongside professionals from other sectors will be essential to preparing them for real-world practice, where implementing solutions to the PH crisis requires coordinated, cross-sectoral teamwork. Ultimately, this will better equip future health professionals to address the health impacts facing patients in an era of environmental disruption. Additionally, stakeholder involvement from organizations setting board exam and curricular standards is also needed for broader PH incorporation. Finally, the survey tool used in this study should be further developed and validated for use in non-US contexts and other health professional schools.

## Conclusion

In conclusion, this study introduced a novel survey and interview tool to assess the integration of PH into medical curricula. Our findings suggest that while student-driven efforts have made significant progress, challenges persist in achieving consistent and systematic PH curricular integration. Key barriers include difficulty sourcing PH content, limited action from instructors and administrators, and resistance to adding content not covered by major medical exams, leading to inconsistent adoption across medical schools. To address this, medical schools should prioritize developing longitudinal, course-specific PH education, supported by both individual institutions and national frameworks and licensing organizations. This commitment is crucial to better equip future healthcare professionals to address environmental health challenges and to advance the broader structural changes needed to build a sustainable healthcare system.

## Data Availability

The raw data supporting the conclusions of this article will be made available by the authors, without undue reservation.
