# Supplementary material for: Student challenges and successes in integration of planetary health in medical education: a mixed methods analysis
Source: Front Public Health. 2025 Jul 24;13:1593332. doi: 10.3389/fpubh.2025.1593332 (PMC12328440; doi:10.3389/fpubh.2025.1593332)
Supplement: Supplementary file 1 [file Table_1.docx]

**Supplementary Table: Demographic Factors of Survey Population (N=31)**

| **Demographics** | **N(%)** |
| --- | --- |
| Respondents | 31 (100) |
| Medical School Class Size  <50  50-100  100-200  >200 | 0 (0)  7 (23)  24 (77)  0 (0) |
| School Type  Public  Private | 16 (52)  15 (48) |
