# Supplementary material for: Student challenges and successes in integration of planetary health in medical education: a mixed methods analysis
Source: Front Public Health. 2025 Jul 24;13:1593332. doi: 10.3389/fpubh.2025.1593332 (PMC12328440; doi:10.3389/fpubh.2025.1593332)
Supplement: Supplementary file 2 [file Data_Sheet_1.pdf]

Intro

Hello there! This survey is coming to you because you have been listed as a contact person for the Planetary Health Report Card (PHRC) at your institution. We're Medical Students for a Sustainable Future (MS4SF). We work alongside organizations including the Planetary Health Report Card (PHRC) and Climate Resources for Health Education (CRHE) to help get planetary health into curricula at health professions schools. We want to better understand some of the challenges (and successes) you've had at your school getting planetary health into the curriculum. Your responses will help us better target resources and workshops toward problem areas. Your responses will be recorded without identifiers and will not affect your standing at your institution, or with MS4SF, CRHE, or PHRC. By proceeding, you are consenting to provide information for this survey

Curricular Reform Leaders

The first questions are about who is leading curricular reform for Planetary Health (PH) at your school. Leading the effort means actively proposing places where PH content could fit in or designing new curricula.

Rank the top 3 stakeholders who are most active in leading the efforts to integrate planetary health content into your school's curricula with 1 being most active. Please leave options after 3 unranked.

- [ ] Deans
- [ ] Course Directors
- [ ] Lecturers/instructors
- [ ] Students
- [ ] Community members
- [ ] Curriculum Committee
- [ ] Other
- [ ] Currently, no one is leading curricular reform

If you indicated other people are leading curricular reform, please specify who (in broad terms, do not use personal identifiers):

Stages of PH curriculum development

What stage is your school at in integrating planetary health content into the curricula?

- Not started integration
- Discussed content integration, but not implemented
- Have integrated PH content
- Have integrated PH content with developing plans to update/review/add more content
- Have integrated PH content with an existing plan for updating/reviewing/adding additional content

Identifying obstacles & successes

Please rate how much you agree or disagree with the following statements:

	Strongly disagree	Somewhat disagree	Neither agree nor disagree	Somewhat agree	Strongly agree
The people leading curricular development are able to identify places to add PH content into your existing curriculum OR ways to modify your existing curriculum to integrate PH.	○	○	○	○	○
Administrators express support for integrating PH content	○	○	○	○	○
Course directors express support for integrating PH content	○	○	○	○	○
Lecturers/instructors express support for integrating PH content	○	○	○	○	○

	Strongly disagree	Somewhat disagree	Neither agree nor disagree	Somewhat agree	Strongly agree
The people leading curricular development are able to find resources to integrate PH content. (e.g., slides, case conferences, learning objectives, curricular outlines/syllabi, elective coursework syllabi)	○	○	○	○	○
The existing resources available (e.g., MS4SF curriculum guide and/or the CRHE resources) can be adapted into your institution's curriculum without much effort.	○	○	○	○	○
There is enough time in the curriculum to teach PH content.	○	○	○	○	○
Instructors feel confident teaching PH content.	○	○	○	○	○

Resources

From where is your school sourcing your planetary health content/materials? Select all that apply.

- ☐ Climate Resources for Health Education (CRHE)
- ☐ Resources created within our institution
- ☐ Resources created by another institution
- ☐ Other

Which institution (do not use personal identifiers).

[ ]

Please elaborate on where your school is sourcing PH content/materials from (do not use personal identifiers).

Demos

How many medical students are in each class/grade of your medical school?

- <50 students
- 50-100 students
- 100-200 students
- >200 students

School type

- Public
- Private

In which state/territory is your medical school located

Follow-up

Would you be willing to be contacted by an MS4SF representative to talk more about your school's experience?

- Yes
- No

You indicated you would like to speak to an MS4SF representative to talk more about your school's experience via Zoom. Note that interviews will be transcribed. Please click this link so we can follow up.

Powered by Qualtrics
